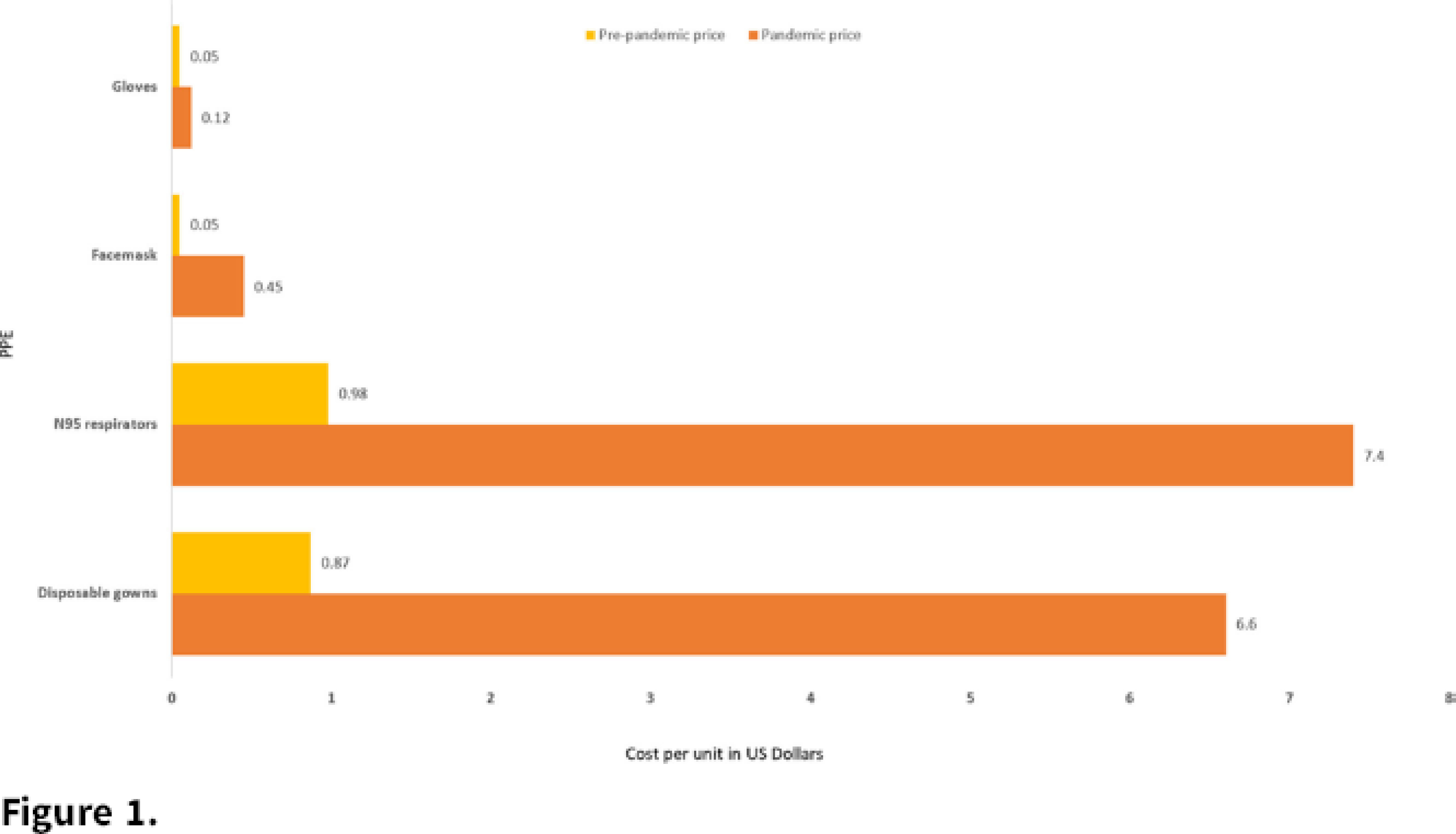# Cost of Personal Protective Equipment During the First Wave of COVID-19

**DOI:** 10.1017/ash.2021.94

**Published:** 2021-07-29

**Authors:** Alfredo Mena Lora, Mirza Ali, Sherrie Spencer, Eden Takhsh, Candice Krill, Susan Bleasdale

## Abstract

**Background:** As the world prepared for and responded to the COVID-19 pandemic in early 2020, a rapid increase in demand for personal protective equipment (PPE) led to severe shortages worldwide. Acquisition of PPE in the general market was an integral part of pandemic response, along with the safeguarding of hospital supplies. We seek to quantify the difference in cost per unit (CPU) of PPE during the first wave of COVID-19 compared to prepandemic prices. **Methods:** We performed a retrospective review of market prices for PPE during the first surge of the pandemic in Chicago. Cost of PPE was tabulated and compared with prepandemic prices. The maximum cost per unit (CPU) of PPE was tabulated for each week, and the average cost throughout the pandemic was calculated. Disposable gowns, washable gowns, N95 respirators, face masks, and gloves were included in our analysis. **Results:** PPE prices were significantly higher during the pandemic compared to prepandemic prices (Figure 1). Disposable gown CPU peaked at $12 during the first week of March, 13.7 times higher than prepandemic prices, and the average gown CPU was 7.5 times higher than prepandemic prices. N95 respirators had a peak CPU of $12, and average CPU was 8 times higher than prepandemic prices. Face-mask CPU peaked at $0.55, 11 times higher, and averaged 9 times higher the regular price. Gloves averaged 2.5 times higher than the prepandemic CPU. **Conclusions:** Market prices for PPE were significantly elevated during the first weeks of the pandemic and remained high throughout the first wave of COVID-19. Multiple factors likely contributed to high prices, including demand shock, disrupted supply chains, and a rush to acquisition by healthcare systems and the general population alike. The impact of COVID-19 on prices highlights the importance of supply chains and national stockpiles for pandemic preparedness.

**Funding:** No

**Disclosures:** None

**Figure 1.**